# Neuropsychological tests and machine learning: identifying predictors of MCI and dementia progression

**DOI:** 10.1007/s40520-025-02962-4

**Published:** 2025-03-12

**Authors:** Carlotta Cazzolli, Marco Chierici, Monica Dallabona, Chiara Guella, Giuseppe Jurman

**Affiliations:** 1 Data Science for Health, Fondazione Bruno Kessler, Via Sommarive 18, Trento, 38123 Italy; 2 Unità Operativa Psicologia, Dipartimento Transmurale Salute Mentale, Azienda Provinciale per i Servizi Sanitari, Viale Verona, Trento, 38123 Italy; 3 Department of Biomedical Sciences, Humanitas University, Milan, 20072 Italy

**Keywords:** Machine learning, Neuropsychological tests, MCI, Dementia

## Abstract

**Background:**

Early prediction of progression in dementia is of major importance for providing patients with adequate clinical care, with considerable impact on the organization of the whole healthcare system.

**Aims:**

The main task is tailoring robust and consolidated machine learning models to detect which neuropsychological tests are more effective in predicting a patient’s mental status. In a translational medicine perspective, such identification tool should find its place in the clinician’s toolbox as a support throughout his daily diagnostic routine. A second objective involves predicting the patient’s diagnosis based on the results of the cognitive assessment.

**Methods:**

281 patients with MCI or dementia diagnosis were assessed through 14 commonly administered neuropsychological tests designed to evaluate different cognitive domains. A suite of machine learning models, trained on different subsets of data, was used to detect the most informative tests and to predict the patient’s diagnosis. Two external validation datasets containing MMSE and FAB tests were involved in this second task.

**Results:**

The tests qualitatively and statistically associated to a cognitive decline are MMSE, FAB, BSTR, AM, and VSF, of which at least three were considered the most informative also by machine learning. 73% average accuracy was obtained in the diagnosis prediction on three subsets of original and external data.

**Discussion:**

Detecting the most informative tests could reduce the visits’ time and prevent the cognitive assessment from being biased by external factors. Machine learning models’ prediction represents a useful baseline for the clinician’s actual diagnosis and a reliable insight into the future development of the patient’s cognitive status.

**Supplementary Information:**

The online version contains supplementary material available at 10.1007/s40520-025-02962-4.

## Introduction

Mild cognitive impairment (MCI) is a transitional state between the cognitive changes due to normal aging and dementia [1, 2]. It is characterized by a noticeable cognitive decline affecting memory, language, and thinking abilities that goes beyond the expected decline caused by biological aging. MCI is considered an intermediate stage between normal age-related cognitive decline and more serious conditions such as dementia or Alzheimer’s disease. Nowadays, approximately 12% to 18% of people older than 60 years old suffer from MCI. Dementia is a progressive neurodegenerative syndrome affecting approximately 55 million people worldwide. Studies show [3] that deterioration of at least two cognitive domains that interfere with semantic memory, executive functions, and, more in general, daily activities is an indicator of dementia. This investigation groups various diseases under the name of dementia (DEM), such as Alzheimer’s disease, frontotemporal dementia, vascular dementia, and dementia with Lewy’s bodies as well as primary progressive aphasia and neurodegenerative disorders. In recent years, life expectancy has increased: in 2000, there were approximately 600 million people over the age of 60 in the world, and it is estimated that this number will increase to 1.2 billion during 2025 and 2 billion in 2050 [4]. As life expectancy increases, the incidence of neurodegenerative diseases characterized by age-related cognitive impairment is bound to increase [5, 6]. Given the high risk of progression from MCI to dementia [7], early identification is therefore crucial for potential targeted therapies aimed at maintaining the patients’ current cognitive status or slowing down the progression to DEM, thus improving the quality of life of the patients and their families. Neuropsychological tests are a key diagnostic tool for assessing MCI and DEM by providing detailed information on different cognitive domains and identifying subtle deficits [8]. They proved to be very useful in diagnosing MCI and tracking the progression of cognitive symptoms over time [1], in particular those tests related to memory, attention, and executive function [9]. Current approaches for predicting the progression from MCI to DEM rely on machine learning, primarily focusing on variables derived from bioimaging such as positron emission tomography (PET) or T1 magnetic resonance imaging (MRI), or extracted from other modalities like electroencephalography and magnetoencephalography, rather than neuropsychological testing variables alone, which are mostly used in aggregated form [10]. In this study, we leverage machine learning algorithms to tackle the task of determining the multivariate predictive value of a battery of 14 neuropsychological tests administered to 281 patients with presumed mental impairment. To the best of our knowledge, few studies assessed the patients on such a comprehensive range of neuropsychological tests. In addition, the relevant available data per patient allow us to perform different tasks. First, we address the issue of selecting the most informative and effective neuropsychological tests to assess the patient’s cognitive state. This represents a major challenge since a small pool of tests may not provide a comprehensive picture of the patients’ mental state while submitting them to an exceedingly wide range of cognitive tasks could produce impaired results as well due to tiredness or progressive lack of self-confidence. Second, this study focuses on whether it is possible to predict a patient’s mental state based only on the results of the cognitive assessment, which is another major challenge: to date, very few studies have presented a machine learning approach to address these issues. In the present study, several Random Forest Classifiers, combined with statistical and qualitative analysis, are trained on different subsets of data and features to provide a binary diagnosis prediction between MCI and DEM. Random Forest (RF) models were previously applied to neuropsychological tests in the context of DEM and MCI, performing well compared to other machine learning methods [11, 12]. The decision-making process of the RF models trained on the battery of all 14 tests is then investigated to understand the relative importance of the tests and integrated with the statistical analysis to determine a ranking of the most informative tests.

As a major outcome, the aforementioned analysis will provide clinicians with additional information on which to base an early and accurate diagnosis. Even though there are no proven treatments to improve the mental conditions of patients suffering from neurodegenerative diseases, an early diagnosis could nevertheless prompt targeted therapies to maintain the current cognitive status and to support patients and caregivers in the acceptance and understanding of the disease.

## Methods

### Patients

All the patients under study were assessed in the Azienda Provinciale per i Servizi Sanitari (APSS) of Trento, Italy between May 2017 and December 2019. All patients gave their written informed consent. All participants were native Italian speakers and none of them had a history of alcoholism or relevant neurological or psychiatric disease. The analysis is based on the results of 14 neuropsychological tests performed by 281 patients. Among them, 133 patients suffered from dementia (DEM), which includes vascular dementia, frontotemporal dementia, Dementia with Lewy Bodies, and Alzheimer’s disease (M = 45% (60 patients), F = 55% (73 patients), age = $76\pm 7$ years, education = $9\pm 4$ years, average ± standard deviation) and 148 patients suffered from Mild Cognitive Impairment (MCI) - a precursor of Alzheimer’s disease - (M = 43% (64 patients), F = 57% (84 patients), age = $76\pm 7$ years, education = $9\pm 4$ years). Due to various reasons, not all of the 281 initial patients participated in the follow-ups, causing the dataset to shrink to 115 patients (68 DEM and 47 MCI) at follow-up 1 (FU1) and subsequently to 51 patients (34 DEM and 17 MCI) at follow-up 2 (FU2). The exclusion criteria were the following:

- Patients regarded as stable by the caregivers;
- Patients who presented serious worsening in a short time period which might led them to move into a nursing home;
- Patients who decided to stop the care pathway because it had become too frustrating;
- Deceased patients.

### Tests

The neuropsychological assessment covered different cognitive functions for a total of 14 tests, each one having a cut-off threshold. The tests are detailed in the following and summarized in Table 1. Global assessment: Mini-Mental State Examination (MMSE); language: naming of nouns (NN), Verbal Fluency on phonemic cue (VPF) and on semantic cue (VSF); memory: Digit Span backward (DSB) and forward (DSF), Corsi span (CS), Babcock story recall test (BSRT), Rey-Osterrieth complex figure delayed copy (ROCF-DC); executive function: Frontal Assessment Battery (FAB), attentional matrices (AM); visuospatial skills: Clock Drawing Test (CDT); praxis: Rey-Osterrieth complex figure copy (ROCF-C); mood: Cornell Scale. A test is deemed passed if the score is greater than or equal to the cut-off threshold, except for the mood test, where the score must be less than the cutoff for the test to be passed, and the Mini-Mental State examination, where the score must be strictly greater than the cut-off for the test to be passed. The duration of the neuropsychological evaluation was approximately 1 h. Parallel versions of some tests were used at first visit (FV) and follow-up (FU), in order to avoid possible learning effects. Raw scores were adjusted for age, education, and gender; specific cut-off values were adopted to evaluate impairments in each test. Adjusted scores allow, for instance, evaluating the net effect of age on patients’ performances. Patients were examined every six months; in fact, after the first visit, they attended follow-up visits, and, according to the disease severity, they could be classified as MCI or DEM. A missing value for a neuropsychological test represents the patient’s inability to complete the test. Given the limited number of patients available for the study, missing values were treated as zeros, which was a consistent interpretation of the patient’s inability to complete the test.

**Table 1:** Test description

Cognitive domain	Cognitive tests	Cut-off
Global cognition	Mini-Mental state examination [13]	$>24$
Language	Naming Nouns [14]	$\ge 8.2$
	Verbal fluency on phonemic cue [15]	$\ge 17.35$
	Verbal fluency on semantic cue [16]	$\ge 25$
Memory	Digit span forward [17]	$\ge 4.26$
	Digit span backward [17]	$\ge 2.65$
	Corsi span [17]	$\ge 3.46$
	Babcock story recall test [16]	$\ge 7.5$
	Rey-Osterrieth complex figure [18]	$\ge 9.47$
Attention and executive	Attentional matrices [19]	$\ge 30$
	Frontal assessment Battery [20]	$\ge 13.4$
Visuo-constructive skills	Clock drawing test [21]	$=1$
Praxis	Copy of Rey-Osterrieth complex figure	$\ge 28.88$
Mood	Cornell scale for depression in dementia [22]	$< 9$

### Statistical analysis

Given the large number of tests each patient underwent and the different ranges of scoring and cut-offs, box plots proved useful in describing the collective situation detected at FV and FUs. In particular, box plots allow us to explore and compare the overall performance of MCI and DEM groups at FV and to better understand how the test scores were affected over time, i.e., from FV to FU2. Provided that fewer patients participated in the follow-ups, the patterns observed at FU1 and FU2 could be influenced by the lack of participation. To quantitatively compare test scores at FV, FU1, and FU2, we used the nonparametric Wilcoxon signed-rank test since nonparametric tests are more suitable with non-normally distributed data such as the neuropsychological test scores. Wilcoxon signed-rank test is the nonparametric counterpart of the t-test and determines whether the mean values of two dependent groups are statistically different from one another [23]. The chosen pairs of groups were the three simple combinations between the test scores at FV, FU1, and FU2 for each diagnosis (thus FV-FU1, FV-FU2, and FU1-FU2) and the p-value calculated from the Wilcoxon test was used to assess the statistical significance of the qualitative trend observed in the box plots. Since CDT values are boolean (true/false), we used mosaic plots for visualization and the Fisher exact test for assessing statistical significance. Fisher exact test is usually applied to small non-parametric groups and determines whether there is a statistically significant association between two categorical variables [24].

### Machine learning analysis

**Fig. 1:**
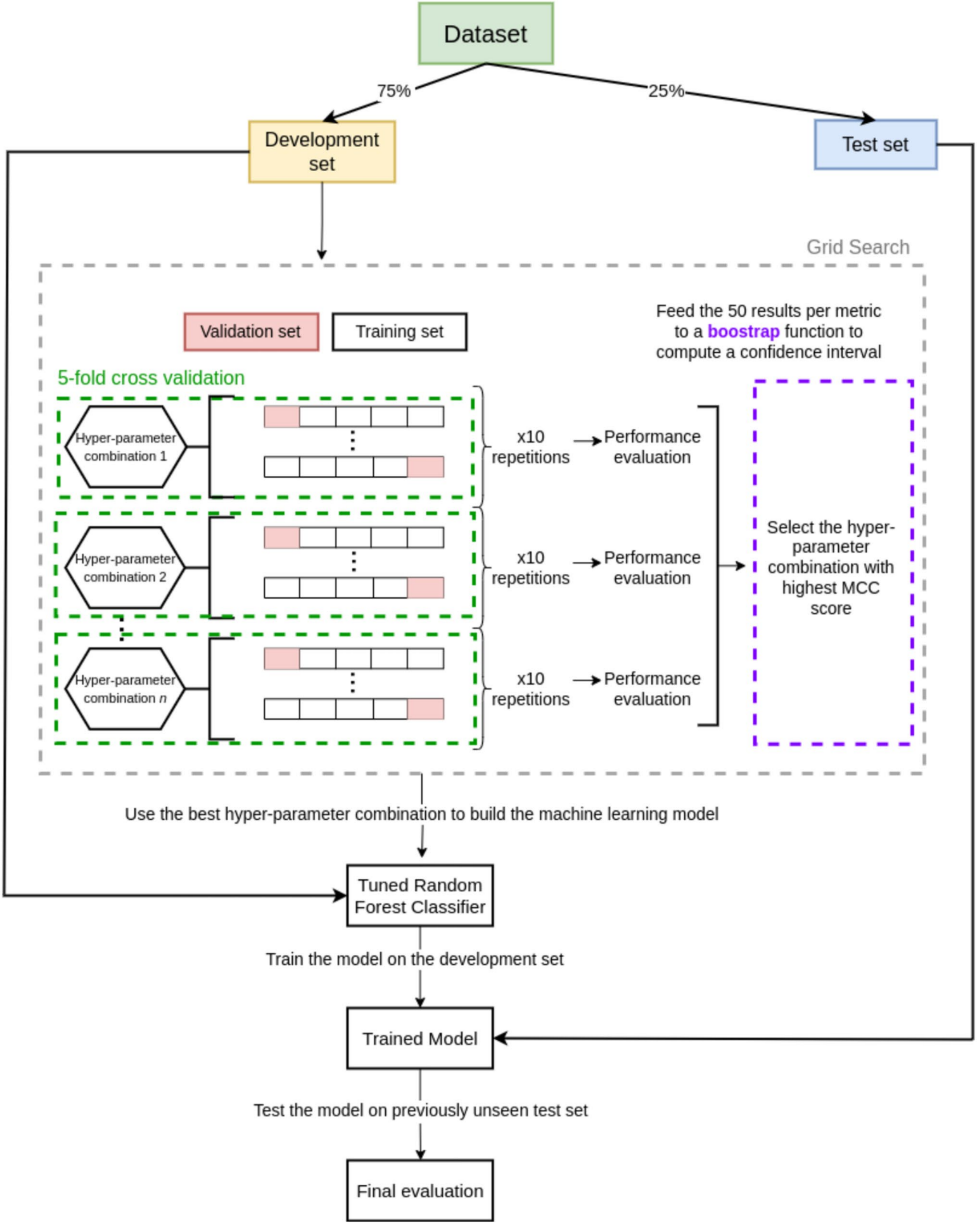
Flow chart representing the machine learning model’s components and their execution

Figure 1 shows a schematic representation of the machine learning analysis workflow. Several Random Forest (RF) classifiers were trained with different subsets of features and labels of the main dataset, to predict the patients’ diagnosis at various stages of the study. RF is a consolidated machine learning algorithm used for classification tasks, which combines the output of multiple decision trees to provide a unique result [25]. Before each training phase, the RF hyperparameters were optimized by an exhaustive grid search, with number of estimators in [25, 50, 100, 150], number of features to consider when splitting a node in [sqrt, $\log _2$, None], maximum depth of the single tree in [3, 6, 9], and maximum number of leaf nodes for each tree in [3, 6, 9]. We evaluated the hyperparameter optimization process by six performance metrics, namely accuracy, Matthews Correlation Coefficient (MCC) [26], precision, recall, F1 score, and the area under the receiver operating characteristic curve (AUC). MCC is a robust statistical measure generally regarded as a balanced performance indicator for binary and multiclass classification, which can be used even when the classes are imbalanced. A stratified 5-fold cross-validation with 10 repeats was implemented for the grid search, and the results for each metric were fed to a bootstrap function in order to compute a 95% confidence interval [27]. The best estimator was then trained on 75% of the given dataset, leaving out 25% for testing. A permutation test was then performed on the best model to investigate the reliability of the computed results. Permutation tests are a statistical method to test the null hypothesis by simulating a consistent number of random arrangements of the data, calculating the statistic of interest (here, MCC), and then comparing the distribution of the permuted statistics with the observed one: if the observed statistic is far from the permuted distribution, one can assume that the null hypothesis may be rejected [28]. The permutation tests were computed using the same training set and RF hyperparameters as the ones used for predicting the diagnosis.

#### First investigation - feature selection

Since submitting elderly people to long cognitive testing could produce biased results due to tiredness over illness, we investigated which tests the RF models deemed important the most during the training process. A total of four RF models were trained for this purpose on two different subsets of the original data.

**Subset 1** For this subset, patients who had valid test scores and diagnosis both at the first examination and at the first follow-up were chosen. The test results that reported a null performance were treated as the patient scored 0 points in that test. The patients who fulfilled the above criteria were 115, of which 68 with MCI and 47 with DEM, and were involved in the development of three tasks: (i) predicting the diagnosis at the first follow-up by training a RF model on the test results collected at the first examination; (ii) predicting the diagnosis at the first examination based on the test results of the first examination; and (iii) predicting the diagnosis at the first follow-up based on the test results of the first follow-up.

**Subset 2** All 281 patients participated successfully in the first examination and were taken into account for this task, regardless if they took part in any follow-up. This data was used in the training and testing of a tuned RF in order to predict the patients’ diagnosis based on their test scores.

#### Second investigation - diagnosis prediction

Two external datasets [29, 30], hereafter referred to as EXT1 and EXT2, respectively, were used to strengthen the RF learning process and predictions of the diagnosis at the first examination based on the test results of the same examination. EXT1 [31] originally included 335 patients (62 healthy aging controls, 186 DEM, 62 MCI, 87 classified as "other disease"). For the purpose of this study, healthy controls and patients with a disease other than MCI/DEM were discarded, thus leaving 248 patients for downstream analysis (F = 145, M = 103; age = 79 ± 6.8, average ± standard deviation). EXT2 [32] originally presented 207 patients (34 healthy controls, 120 DEM, 53 MCI). After removing healthy controls, 173 patients are available for downstream analysis (F = 97, M = 76; age = 78 ± 6.9). EXT2 also presented features from magnetoencephalography, which were also discarded. The patients in EXT2 underwent the neuropsychological assessment at the Kumagaya General Hospital between 2019 and 2021 and were diagnosed by a neurosurgeon and clinical instructor at the Japanese Society of Dementia Research. Likewise, the patients in EXT1 underwent the neuropsychological assessment at the Kumagaya General Hospital. Both datasets included MMSE and FAB as features, with distributions compatible with our original data (OD) and a binary diagnosis classification divided between MCI and DEM. The processing of the OD consisted of discarding all the tests except for MMSE and FAB so that the merged datasets would contain the two tests as features and the diagnosis as label. Three classifiers were trained on three different combinations of the available data: two datasets were obtained by merging OD with the EXT1 and EXT2 separately (529 and 454 patients, respectively), and the third dataset included OD and the two external datasets together (702 patients).

## Results

### Statistics

**Snapshot of first examination** Over 75% of MCI patients scored above the cut-off in VSF, VPF, DSF, DSB, CS and AM tests, MMSE and ROCF-C tests were successfully completed by over 50% of participants and only NN and CSD plots were entirely above the cut-off line. A test was considered deficient when over 50% of patients scored below the cut-off: 50–75% of MCI patients were deficient in BSRT, ROCF-DC, and FAB tests. In general, DEM patients performed worse in each task, and none of the tests was successfully completed by all participants (outliers excluded), as happened in MCI patients with NN and CSD tests. Over 75% of DEM patients scored above the cut-off in NN, DSF, DSB, CS, and CSD, while 50–75% of patients successfully passed VSF, VPF, AM, and ROCF-C tests. As opposed to MCI patients, 50-75% of DEM patients proved deficient in the MMSE test; the same was found with the BSRT test. While none of the MCI distributions went below the cut-off of more than 75%, this is the case of ROCF-DC and FAB tests in DEM patients.

**Fig. 2:**
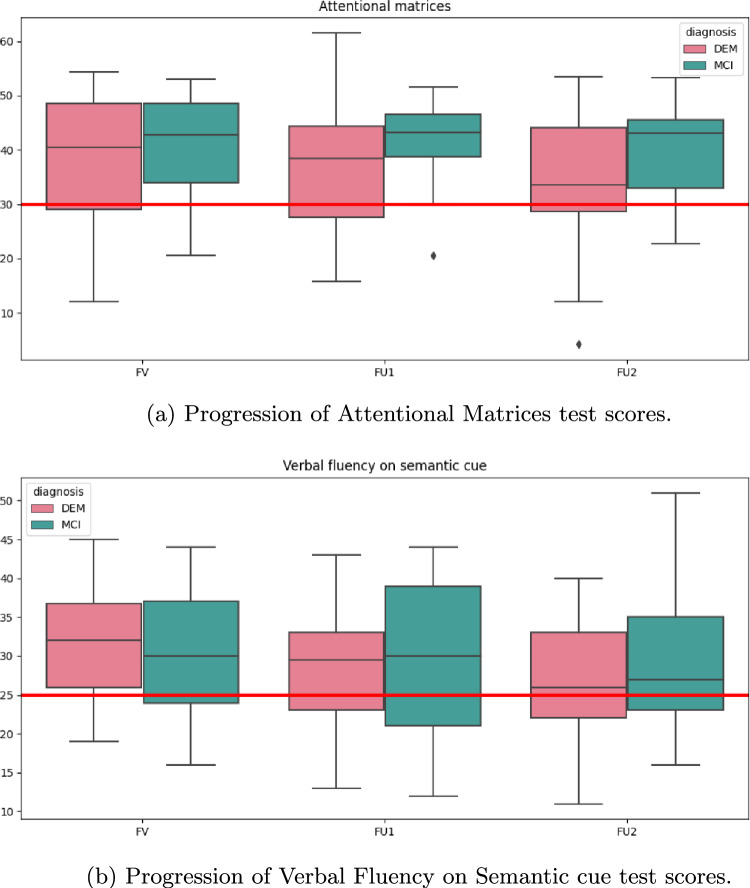
Boxplots showing qualitative progression of test scores from first examination (FV) through follow-ups 1 and 2 (FU1, FU2)

**FUs progression** Among the 14 tests, five of them better underlined the neuropsychological decline of patients affected with dementia as opposed to MCI patients, who have proven to remain stable through the FUs. From a qualitative perspective, DEM patients’ test scores gradually worsen over time in MMSE, FAB, BSTR, AM (Fig. 2a), and VSF tests (Fig. 2b). Wilcoxon’s computed p-values demonstrate a significant worsening of VSF among DEM patients from FV to FU1 ($p=0.02$), from FU1 to FU2 ($p=0.002$), and from FV to FU2 ($p<10^{-3}$), as well as AM among DEM patients from FV to FU2 ($p=0.02$). Fisher test did not highlight significant results.

### Machine learning

#### First investigation - feature selection

The feature importance function applied on each trained RF classifier ranked the features from the one the RFs relied on the most to make their classification to the less informative test. The five most significant tests to assess a patient’s cognitive status for each RF model are shown in Table 2.

**Table 2:** Top-ranked features

FV$\rightarrow$ FU1	FV $\rightarrow$FV	FU1$\rightarrow$ FU1	FV $\rightarrow$ FV^1^
ROCF-DC	FAB	MMSE	VSF
FAB	DSB	FAB	FAB
MMSE	VSF	AM	BSRT
VSF	BSRT	VPF	MMSE
BSRT	MMSE	DSB	ROCF-C

Using the whole dataset

Interestingly, some tests, such as MMSE, FAB, VSF, and BSRT, are recurrent in all top-five lists.

#### Second investigation - diagnosis prediction

The grid search for the best hyperparameters (Online Resource, Table S1) provided 50 CV results for each metric. This data was fed to a bootstrap function to obtain a confidence interval, which consists of a lower and an upper bound between which the mean value of the CV results is 95% likely to be found. Table 3 shows both the mean value of the CV results and the computed confidence interval and the classification performance of the three RF models computed on a previously unseen test set. The model trained on OD+EXT1+EXT2 reaches the highest CV performance, followed by the model trained on OD+EXT1. The different performance of the models trained on OD+EXT1 and OD+EXT2 was not due to demographic differences between EXT1 and EXT2 (age: p-value 0.269, Mann–Whitney U test; gender: p-value 0.624, Z test for proportions) and is likely related to the sample size of the datasets: in fact, the models’ performance tends to increase in all the metrics scores when the amount of training data is larger, as highlighted by the results of the model trained on OD+EXT1+EXT2, which outperforms all the other models, likely due to the increased amount of available data.

**Table 3:** Model performance in cross-validation with grid search (CV) and on the test set (Test)

	Metric	OD+EXT1	OD+EXT2	OD+EXT1+EXT2
CV	Accuracy	0.72 (0.70–0.73)	0.67 (0.66–0.69)	0.76 (0.75–0.77)
	Precision	0.75 (0.74–0.76)	0.70 (0.69–0.72)	0.79 (0.78–0.80)
	Recall	0.76 (0.74–0.77)	0.72 (0.64–0.74)	0.84 (0.83–0.86)
	F1-score	0.75 (0.74–0.76)	0.72 (0.69–0.72)	0.81 (0.80–0.82)
	MCC	0.42 (0.40–0.46)	0.34 (0.32–0.37)	0.47 (0.45–0.49)
	AUC	0.77 (0.76–0.78)	0.73 (0.72–0.75)	0.82 (0.82–0.83)
Test	Accuracy	0.74	0.72	0.72
	Precision	0.80	0.79	0.75
	Recall	0.82	0.70	0.82
	F1-score	0.81	0.74	0.78
	MCC	0.36	0.44	0.39
	AUC	0.76	0.72	0.69

For CV, metric values are expressed as mean with bootstrapped 95% confidence intervals

* MCC* Matthews Correlation Coefficient, *AUC* area under the receiver operating characteristics curve, *OD* original dataset, *EXT(1,2)* external dataset (1,2)

Permutation test results (Online Resource, Fig. S1) showed a significant departure between the MCC score results computed on the data with original labels and the ones computed on random arrangements of the data labels. In permutation tests, small p-values suggest that there is a real dependency between features and targets which has been used by the RF models to give robust predictions. In all three cases, p-values were $\approx 0.0099$, confirming that our models performed significantly better than a random classifier.

## Discussion

Since neuropsychological diseases such as Mild Cognitive Impairment and Dementia affect tens of millions of people worldwide, this investigation aimed to provide practical means and knowledge to early diagnose these diseases and subsequently improve the lifestyle and treatment of patients. The predictive analysis of cognitive tasks for detecting or anticipating dementia yielded significant insights, emphasizing the informative potential of specific neuropsychological tests such as MMSE, FAB, BSTR, AM, and VSF. The qualitative and statistical analysis, which included a Random Forest model, revealed these tests to be crucial markers of cognitive decline over time. The identification of the above tests as key indicators of dementia progression highlights the importance of their prioritization in clinical assessments. Performing these tests at the beginning of a cognitive assessment visit can contribute to observing a less impaired performance by external factors.

Organizing the neuropsychological assessment visit in order to submit the most informative tests before the others can bring benefits to both patients and clinicians. This prioritization presents an opportunity to accelerate the diagnostic process and to eliminate less impactful tests from the battery if the patient shows a significantly impaired performance in the first part of the assessment.

In fact, since neuropsychological patients are often elderly people in a fragile mental state due to their illness, reducing the visits’ load could bring positive feedback from the patients, who are less likely to feel tired or frustrated at the end of the cognitive assessment and are therefore more likely to proceed in the follow-ups. Moreover, tiredness and progressive lack of motivation and self-confidence could reflect in the performance of the tests as the clinician proceeds with the assessment; therefore, submitting to the patients the most informative tests first could lead to a more accurate, unbiased, and plausible diagnosis.

Similarly to recent work by Park and colleagues [33], the present study also relies on multiple neuropsychological tests to detect trends in cognitive function over time. However, here we propose not to use a multi-domain composite index to analyze the results nor to rely on preconstructed test batteries that provide a single score for the analysis of different cognitive functions (e.g., Preclinical Alzheimer’s Cognitive Composite, PACC, or Alzheimer’s Disease Assessment Scale, ADAS13), but to determine the results for each test individually. This makes it possible to highlight the cognitive function actually involved in the decline and then plan targeted cognitive stimulation based on the test results. Moreover, the analysis proposed in this investigation strongly relies on models’ explainability, i.e., the possibility of examining which features influenced the algorithm decision the most and extracting meaningful insights from this information, such as which are the most representative tests to assess one’s neuropsychological status. Overall, one key innovation of this study is the extensive list of neuropsychological tests administered to the patients, usually limited to a battery of few tests. Relying on such comprehensive assessments made it possible to analyze various aspects of the disease progression and the affected cognitive abilities and to systematically evaluate the contribution of each test in effectively determining a patient’s cognitive status.

The classification results are a promising starting point to predict a patient’s diagnosis based on their test score. However, given the wide battery of neuropsychological tests, it would be beneficial to work with a more comprehensive number of patients and to assess the disease progression over a longer period of time, ideally 36 months, maintaining a constant number of participants in the follow-ups. For future developments it could also be beneficial to expand the territorial coverage of the study to allow people coming from different places to participate in the assessment: this could help level cultural and educational biases created by enrolling people with similar backgrounds. Nevertheless, the classification scores achieved by the present study could represent a valid and reliable baseline for supporting clinicians in the diagnostic process and providing them with a second opinion in dubious situations. The additional level of confidence given by the model’s prediction could provide clinicians with a clearer and more thorough view of the patient’s cognitive state and allow them to take practical measures even after the first visit. Being able to promptly and consistently prepare families and caregivers on what behaviors to expect due to the patient’s illness could result in a significant improvement in the lifestyle of both parties. Since caregivers will daily witness the disease progression, providing them with psychological support and practical means to assist the patients could help them better comprehend what a neurodegenerative disease entails and how to properly address people affected by it.

Based on the predicted diagnosis, clinicians have the means to decide how to schedule follow-ups in an efficient and sensible way. This is of significant concern, as patients may find neuropsychological assessments frustrating, and their caregivers may perceive them as less primary in comparison to other medical visits. Strategic follow-up planning can avoid submitting moderately stable patients to too frequent cognitive testing in order not to give them a distorted perception of the gravity of their illness: an ill-considered visit scheduling could undermine the patients’ self-confidence and could result in test outcomes compromised due to anxiety.

Neurodegenerative diseases can not be cured, and they are likely to worsen over time. Timely diagnosis with reasonable certainty allows clinicians to suggest targeted exercises to help conserve the current ongoing situation. Early diagnosis can also be useful for planning suitable therapy, in combination with individual or group cognitive stimulation sessions.

## Conclusion

Mild Cognitive Impairment and Dementia are widespread diseases in elderly people and early diagnosing and treating them is increasingly becoming a major concern. Despite the fact that this study focused on a small group of patients with limited background diversity, who participated in a non-optimal number of follow-ups, the obtained results are encouraging and promising. Applying machine learning to predict the patient’s diagnosis from commonly administered neuropsychological tests is an innovative technology with great improvement potential and a wide range of possible applications to improve MCI and Dementia patients’ life conditions and treatment.

## Supplementary Information

Below is the link to the electronic supplementary material.

Supplementary file 1 (pdf 187 KB)

## Data Availability

The data that support the findings of this study are not openly available due to reasons of sensitivity and are available from the corresponding author upon reasonable request.
