# Supplementary material for: Neuropsychological tests and machine learning: identifying predictors of MCI and dementia progression
Source: Aging Clin Exp Res. 2025 Mar 12;37(1):79. doi: 10.1007/s40520-025-02962-4 (PMC11903588; doi:10.1007/s40520-025-02962-4)
Supplement: Supplementary file 1 — (pdf 187 KB) [file 40520_2025_2962_MOESM1_ESM.pdf]

Neuropsychological tests and machine learning: identifying predictors of MCI and dementia progression

Carlotta Cazzolli[1], Marco Chierici[1], Monica Dallabona[2], Chiara Guella[2], Giuseppe Jurman[1*]

[1*]Data Science for Health, Fondazione Bruno Kessler, Via Sommarive 18, Trento, 38123, Italy.

[2]Unità Operativa Psicologia, Dipartimento Transmurale Salute Mentale, Azienda Provinciale per i Servizi Sanitari, Viale Verona, Trento, 38123, Italy.

*Corresponding author(s). E-mail(s): giuseppe.jurman@fbk.eu; Contributing authors: ccazzolli@fbk.eu; chierici@fbk.eu; monica.dallabona@apss.tn.it; chiara.guella@apss.tn.it;

Supplementary material

Data preprocessing

The data preprocessing was structured as follows: among the three results for each neuropsychological test (raw, corrected, and equivalent), only the corrected score was analyzed since it is adjusted according to the demographic status of the patient and therefore provided a more complete picture of the patient's mental status. Not-a-number values and missing values were replaced with 0 according to the explanation provided in Section 2.2 ("Tests"). Different diagnoses were grouped as follows:

- MCI (Mild Cognitive Impairment): mild cognitive impairment, amnestic mild cognitive impairment, mild cognitive deficit, early cognitive deficits, subjective memory disorder.
- DEM (Dementia): vascular dementia with behavioral disturbances, Alzheimer's disease, vascular dementia, frontotemporal dementia with behavioral disturbances, degenerative dementia, frontotemporal dementia, mixed dementia, mild

dementia, primary progressive aphasia, cerebral degeneration, dementia, and dementia with Lewy bodies.

Patients with diagnosis of Parkinson's disease (3), Mood Disorder (9) were excluded from the study since their condition was not comparable neither to MCI nor DEM. Moreover, undiagnosed patients were also excluded. This process shaped the 281 patients dataset described in section 2.1 ("Patients"). Finally, binary labels were assigned to the diagnosis of MCI (0) and DEM (1) for the purpose of classification.

Random Forest Classifier

The machine learning workflow was implemented in Python 3.10 and it was built on top of the *scikit-learn* 1.3.0 library [1]. The following table reports the optimal hyper-parameters for each Random Forest model.

Table S1: Best hyper-parameters for each Random Forest Classifier

	Datasets		
Metrics	OD+EXT1	OD+EXT2	OD+EXT1+EXT2
Maximum depth	3	9	9
Maximum number of features	sqrt	sqrt	$\log_2$
Maximum leaf nodes	9	3	9
Number of estimators	150	25	25

Permutation tests

The `permutation_test_score` function of *scikit-learn* [1] was used to assess the significance of the models' performance through a permutation test. The function takes in input the model, the data to fit with the corresponding labels, a cross-validation object, and the scoring criteria, which in this case was the Matthews Correlation Coefficient. The test works by first computing the score of the model on the given data and then shuffling the labels 100 times and calculating the corresponding scores to create a null distribution. In the end, a comparison between the actual score with the one of the null distribution is made in order to compute a p-value, i.e., the probability of obtaining by chance the same performance on the null distribution and on the actual score. The function returns the model's score on real data, the scores from the permuted data sets, and the p-value. For reproducibility, randomness was controlled by setting the argument `random_state`=30.

Bootstrapped confidence intervals

The `bootstrap` function (*bootstrapped* Python module) was given in input the results of the cross-validated metrics related to the best model, i.e. the model with the hyper-parameters listed in table S1 and a *stat_func* parameter, which in this case was the mean. The algorithm works by randomly resampling the passed scores $10,000$ times, calculating the mean on each resampled dataset, and building a possible distribution of

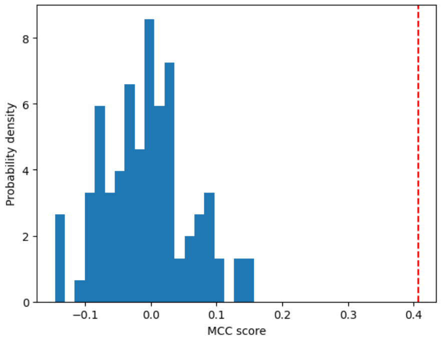

(a) Original dataset combined with EXT1.

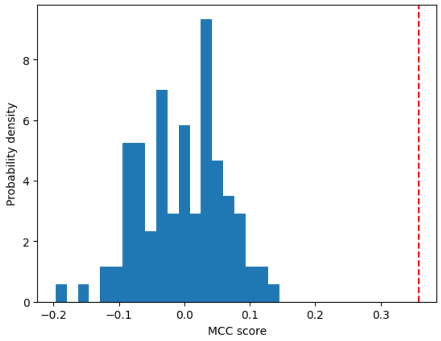

(b) Original dataset combined with EXT2.

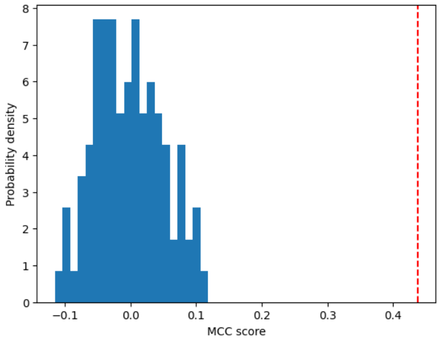

(c) Original dataset combined with EXT1 and EXT2.

Figure S1: Histograms of the permutation test MCC scores. Red dashed lines indicate the classifier's performance on data with unshuffled labels.

means. This process allows for the creation of a 95% confidence interval, providing an estimate of the uncertainty around the mean. This technique is used to obtain a more reliable estimate of the performance and helps ensure that the results are statistically rigorous.

References

[1] Fabian Pedregosa, Gaël Varoquaux, Alexandre Gramfort, et al. Scikit-learn: Machine learning in Python. *Journal of Machine Learning Research*, 12(85):2825–2830, 2011.
